# Governor Brown Turned down the Dentists' Bill

**Published:** 1894-07

**Authors:** 


					138 American Journal of Dental Scienci
Editorial, Etc.
Governor Brown, of Maryland.
turned down the den-
tist's bill, because of its incompletness.?Maryland Medical
Journal.

				

